# Supplementary material for: Pontine Waves Accompanied by Short Hippocampal Sharp Wave-Ripples During Non-rapid Eye Movement Sleep
Source: Sleep. 2023 Jul 21;46(9):zsad193. doi: 10.1093/sleep/zsad193 (PMC10485565; doi:10.1093/sleep/zsad193)
Supplement: zsad193_suppl_Supplementary_Materials [file zsad193_suppl_supplementary_materials.pdf]

Supplementary Information

Pontine waves accompanied by short hippocampal sharp wave-ripples during non-rapid eye movement sleep

Tomomi Tsunematsu[1,2], Sumire Matsumoto[2,4], Mirna Merkler[3,4], and Shuzo Sakata[3*]

[1] Department of Integrative Life Sciences, Graduate School of Life Sciences, Tohoku University, Sendai 980-8577, Japan

[2] Frontier Research Institute for Interdisciplinary Sciences, Tohoku University, Sendai 980-8578, Japan

[3] Strathclyde Institute of Pharmacy and Biomedical Sciences, University of Strathclyde, 161 Cathedral Street, Glasgow G4 0RE, UK

[4] These authors contributed equally.

* Correspondence: shuzo.sakata@strath.ac.uk

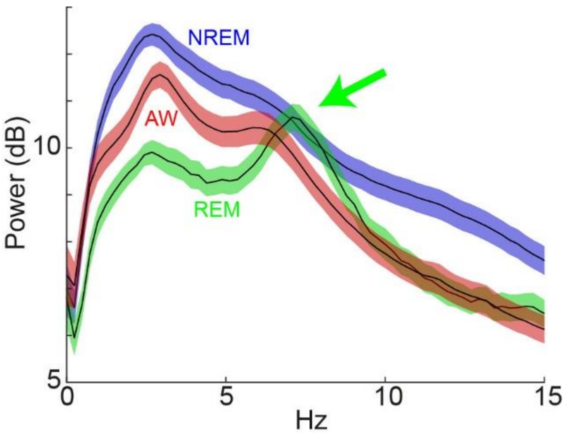

Supplementary Figure 1. Average power spectral density in each state.

High theta (~7 Hz) power was apparent during REM sleep (*arrow*). Note that due to the variability of low-cut filter settings across experiments, low frequency (<1 Hz) power did not reflect real signals. AW, wakefulness; NREM, NREM sleep; REM, REM sleep. Errors indicate SEM.

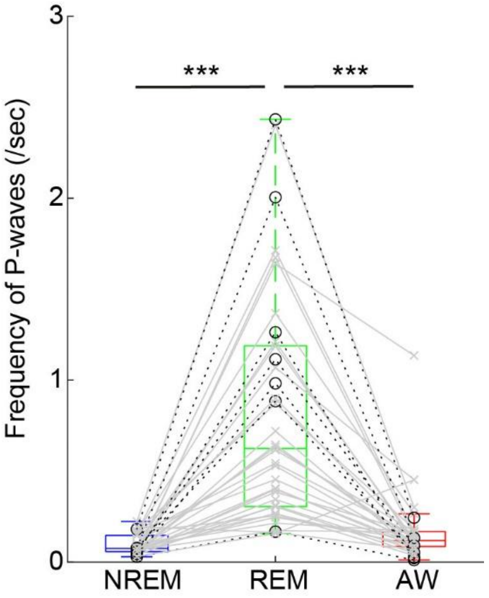

Supplementary Figure 2. P-wave frequency across the sleep-wake cycle.

Circle, silicon probe recording. *Cross mark*, bipolar electrode recording. ***, $p < 0.0001$ (Kruskal-Wallis test with post-hoc Bonferroni method).

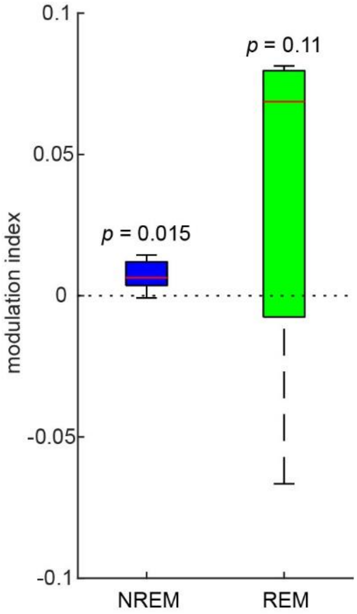

Supplementary Figure 3. **P-wave-associated calcium transients in mesopontine cholinergic neurons.**

The modulation index was defined as $(F_{post} - F_{pre})/(F_{post} + F_{pre})$, where F_{pre} and F_{post} are normalized fluorescent signals 1 s before and after P-wave timing, respectively. Wilcoxon signed-rank test was performed to examine if the median is zero.

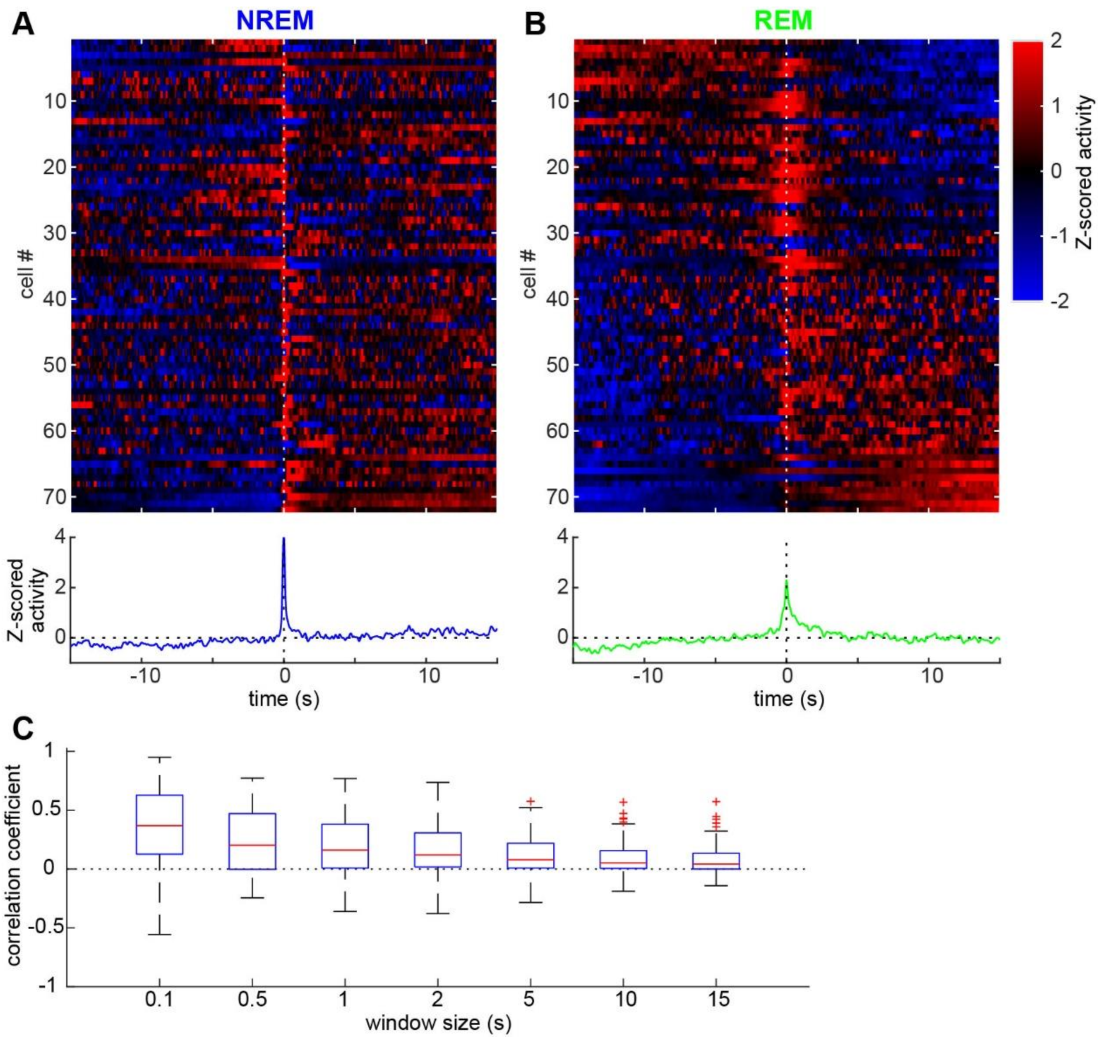

Supplementary Figure 4. **P-wave-associated brainstem neural ensemble dynamics on a long timescale.**

Brainstem neural activity during NREM sleep (**A**) and REM sleep (**B**). *top*, heat map of peri-event time histograms across recorded neurons. Neurons were sorted by the first principal component of the heat map during REM sleep. Individual neuronal activity was Z-scored. *bottom*, the average of Z-scored activity. (**C**) Pearson's correlation coefficient at different window sizes. As the window size increases, the correlation coefficient decreases ($p < 0.0001$, Kruskal-Wallis test).

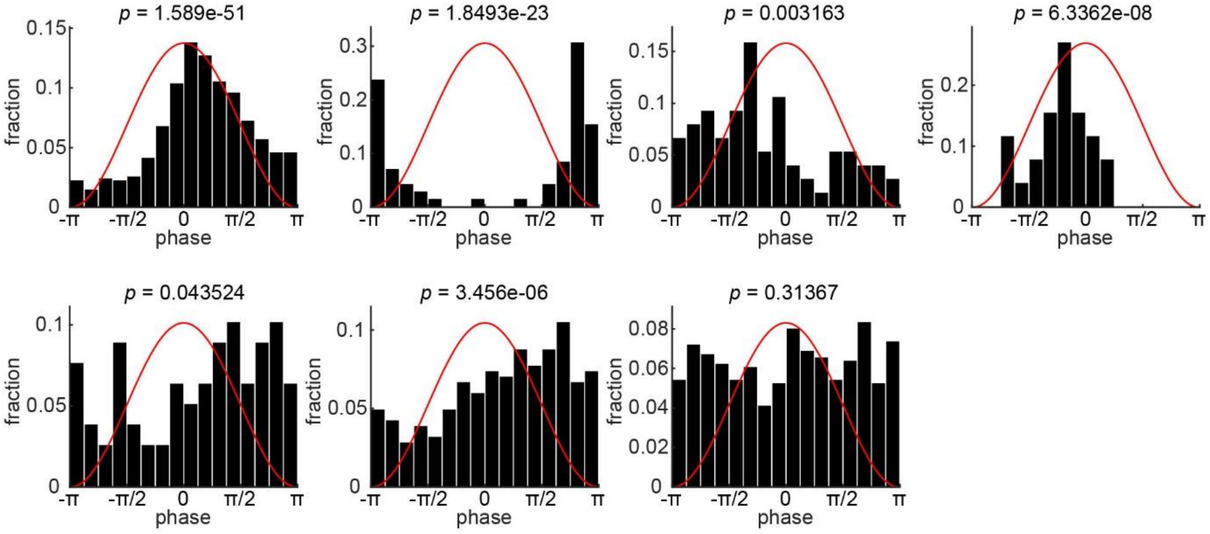

Supplementary Figure 5. Theta-P-wave coupling during REM sleep.

Phase histograms of P-wave timing relative to hippocampal theta rhythms across recordings (n = 7). *P*-values, Rayleigh's test. *Red line*, a theta cycle (4 – 10 Hz).

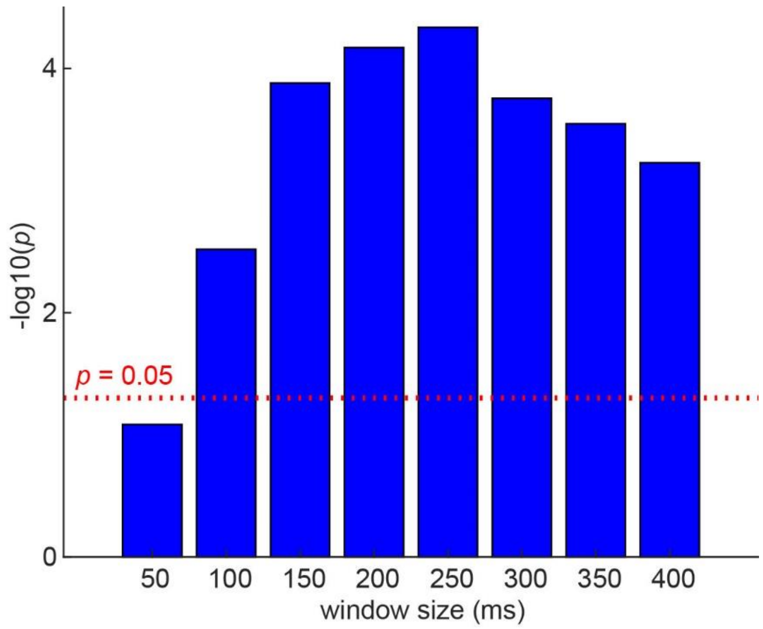

Supplementary Figure 6. The effect of window size on ripple power change after P-waves.

Related to **Figure 4E**, different window sizes were examined. After calculating the asymmetry index, *t*-test was performed to examine if the mean is zero. P-values were shown as a function of window size.

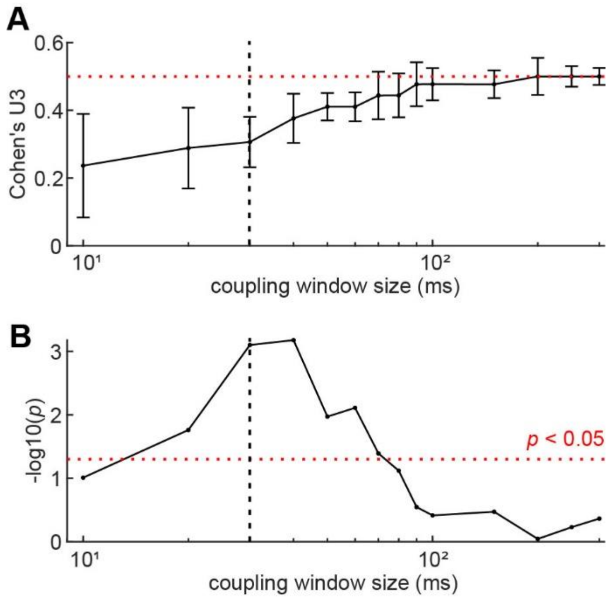

Supplementary Figure 7. The relationship between coupling window size and statistical assessment.

(**A**) Cohen's U3 as a function of coupling window size. Errors indicate 95% confidence intervals. (**B**) *P*-values (rank-sum test) as a function of coupling window size.

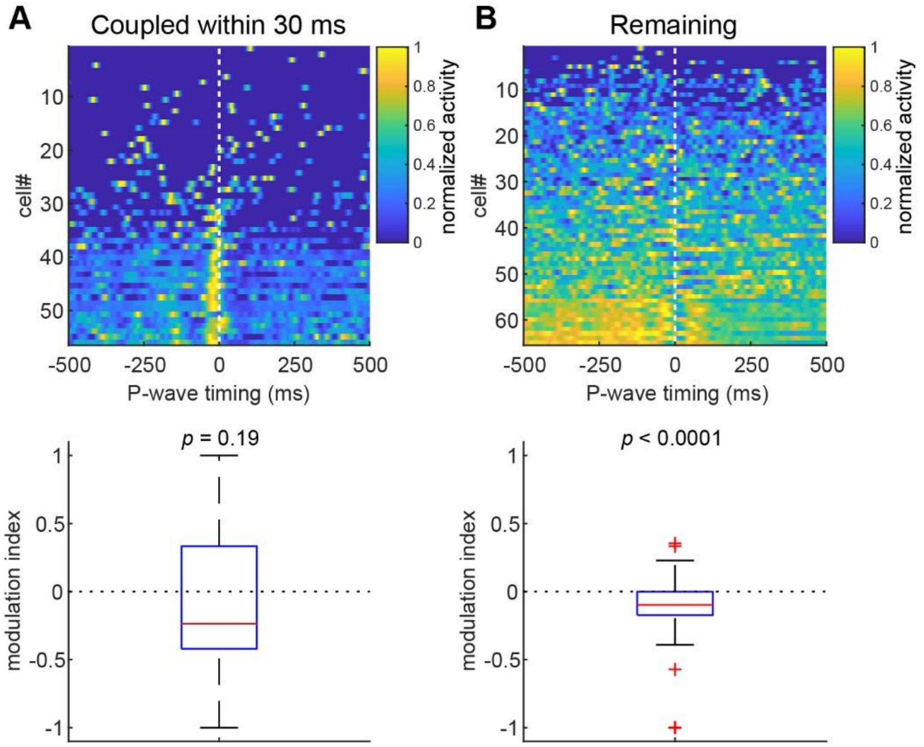

Supplementary Figure 8. CA1 firing at P-wave timing depending on SWR coupling.

CA1 firing at P-wave timing was separated depending on coupling to SWRs within a 30 ms (**A**) or longer time window (**B**). *top*, heat map of normalized peri-event time histograms across neurons. Individual neural activity was normalized by peak firing rate to 1. Neurons were sorted by the first principal component in each heat map. *bottom*, the modulation index was defined as $(FR_{post} - FR_{pre})/(FR_{post} + F_{Rpre})$, where FR_{pre} and FR_{post} are normalized firing rate 250 ms before and after P-wave timing, respectively. *t*-test was performed to examine if the mean is zero.

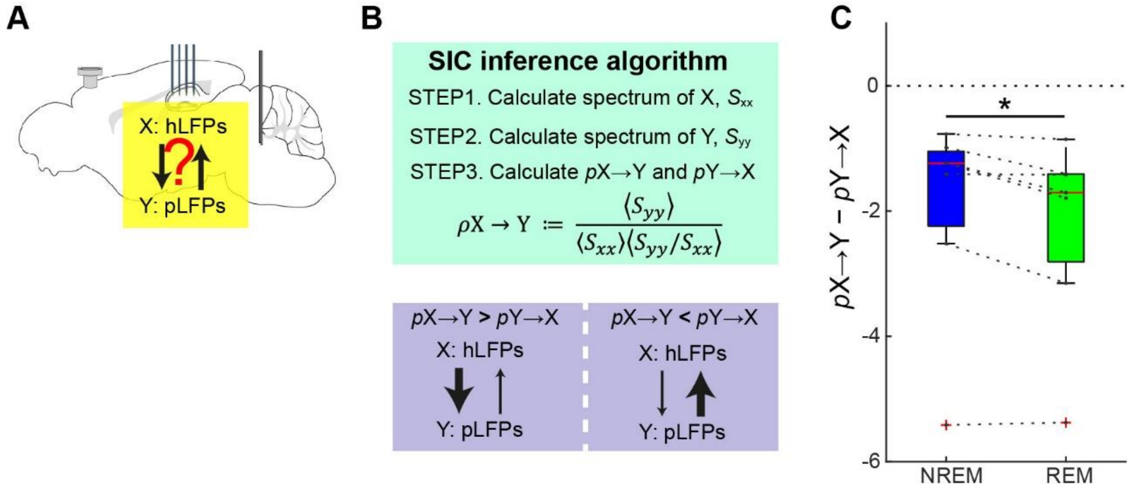

Supplementary Figure 9. Spectral Independence Criterion analysis between hippocampal and pontine LFPs.

(**A**) A schematic drawing outlines the motivation for this analysis. (**B**) An algorithm to compute the spectral density ratio based on the Spectral Independence Criterion (SIC). The interpretation of the outcome is also shown at the bottom. (**C**) The difference between *p*X→Y and *p*Y→X. The smaller value implies a stronger causal direction from pontine to hippocampal LFPs. *, $p < 0.05$ (signed rank test).
